# Bilateral Superior Semicircular Canal Dehiscence Concurrent With Ehlers-Danlos Syndrome: A Case Report

**DOI:** 10.7759/cureus.19943

**Published:** 2021-11-27

**Authors:** Ansley Unterberger, Jessa Miller, Quinton Gopen, Isaac Yang

**Affiliations:** 1 Neurosurgery, David Geffen School of Medicine, University of California, Los Angeles, Los Angeles, USA; 2 Otolaryngology - Head and Neck Surgery, University of California, Los Angeles, Los Angeles, USA; 3 Otolaryngology - Head and Neck Surgery, David Geffen School of Medicine, University of California, Los Angeles, Los Angeles, USA

**Keywords:** case series, ehlers-danlos syndrome, hypermobility, middle fossa craniotomy, superior semicircular canal dehiscence

## Abstract

Superior semicircular canal dehiscence (SSCD) is characterized by temporal bone thinning, which creates an opening between the inner ear and middle cranial fossa. Ehlers-Danlos syndrome, hypermobility type (EDS-HT) is a genetic collagen synthesis disorder, often resulting in bony abnormalities. We present the case of a 39-year-old female with EDS-HT who exhibited the otological symptoms characteristic of bilateral SSCD. High-resolution computed tomography (CT) scans confirmed the diagnosis. The patient elected for middle fossa craniotomy and noted symptomatic improvement. Due to its potential to confer bone fragility, EDS-HT may predispose SSCD development. Further examination of the relationship between these disorders is necessary.

## Introduction

Superior semicircular canal dehiscence (SSCD) is characterized by tegmen tympani thinning, which creates a third opening between the inner ear and middle cranial fossa [1,2]. This pathological window changes cochlear fluid flow, alters air and bone conduction thresholds, and engenders vertigo, disequilibrium, tinnitus, and hyperacusis [2-4]. To diagnose this syndrome, physicians employ vestibular-evoked myogenic potentials (VEMPs) [4,5], audiometric testing, and high-resolution temporal computed tomographic (CT) scans [2,4]. The preferred surgical technique is via the middle cranial fossa, as it provides greater dehiscence visualization than the transmastoid approach [6,7]. Ehlers-Danlos syndrome hypermobility type (EDS-HT) is a subset of the aforementioned connective tissue disorder and is defined by joint hypermobility and instability [8]. Dolan et al. reported that compared to age-matched controls, EDS patients more frequently experienced bone formation abnormalities [9]. We postulate that EDS-HT may predispose individuals to SSCD. Previously, our laboratory described two patients [10,11] who suffered from concurrent bilateral SSCD and EDS-HT. In this study, we present the case of a third patient, a 39-year-old female with similar diagnoses, and compare her course to our previously reported cases. Due to the rarity of EDS-HT patients with concurrent SSCD, there is a paucity of such patients described in the literature. As such, our aim is to amalgamate such cases in one manuscript to help define the correlation between SSCD and EDS-HT. All patients were treated by the same neurosurgeon and otolaryngologist in one institution.

## Case presentation

History and examination

**Figure 1:**
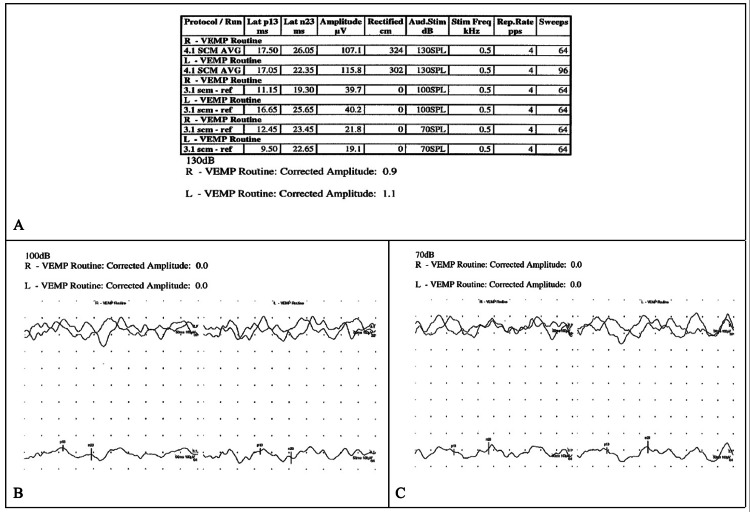
A 39-year-old woman presenting with many auditory and vestibular symptoms including hyperacusis, tinnitus, and vertigo. Routine VEMP revealing corrected amplitudes for left (L) and right (R) ears at A) 130 dB, B) 100 dB, and C) 70 dB VEMP, vestibular-evoked myogenic potential

**Figure 2:**
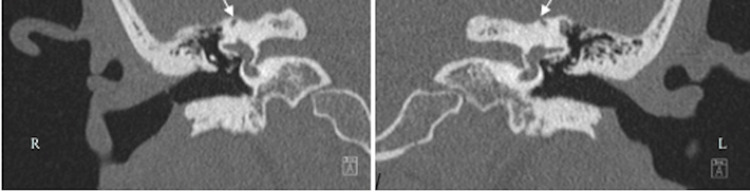
A 39-year-old woman presenting with many auditory and vestibular symptoms including hyperacusis, tinnitus, and vertigo. High-resolution CT scan shows thinning of the (R) right and (L) left osseous roof of the bilateral semicircular canals with possible areas of dehiscence. CT, computed tomography

A 39-year-old female with a past medical history of Ehlers-Danlos Syndrome, postural orthostatic tachycardia syndrome (POTS), and chronic migraines presented with a 1.5-year history of left greater than right-sided disequilibrium after an ice-skating fall. She also reported right greater than left-sided pulsatile tinnitus, autophony, dizziness, vertigo, bilateral internal sound amplification, and aural fullness. VEMP results at an outside institution demonstrated slightly reduced right- and left-sided threshold down to 80 dB and 85 dB, respectively. At our institution, we found right-sided VEMP, corrected amplitude of 0.9 (130 dB), 0.0 (100 dB), and 0.0 (70 dB), and left sided, 1.1 (130 dB), 0.0 (100 dB), and 0.0 (70 dB) (Figure 1A-1C). Air and bone conduction were within normal limits. Pure tone thresholds were normal. Speech reception threshold indicated 5 dB and 0 dB in her right and left ears, respectively. Her word recognition score was 100% at 50 dB, bilaterally. CT revealed bilateral semicircular canal osseous roof thinning with possible dehiscence (Figure 2).

Surgery and follow-up

Because the patient was chiefly concerned by her left-sided dizziness coupled with the fact that CT imaging demonstrated a larger left-sided defect, the patient first underwent a left-sided middle fossa craniotomy via a keyhole preauricular infratemporal approach. After positioning, surgeons reconstructed preoperative CT scan images to identify coordinates for BrainLab neuronavigation (Munich, Germany). During the surgery, facial nerve, brainstem auditory-evoked responses, baseline SSEP, and EEG-evoked potentials were monitored with needles in their proper locations. Middle cranial fossa floor microdissection identified the SSCD. Using a moist microcottonoid, surgeons secured warm bone wax to the SSCD and inserted harvested bone from the initial craniectomy within the wax. Heliostat matrix, followed by fibrin sealant, were applied over the surgical area. The craniotomy was closed in the standard procedure. There were not changes in the intraoperative monitoring of the VIII and VII cranial nerves. There were no postoperative complications, and the patient was discharged in stable condition. Six days postoperatively, she noted mild improvement in auditory and vestibular symptoms, but reported left eyebrow movement impairment, dizziness, nausea, and heartbeat internal sound amplification when lying on her left side. During her six- and nine-week follow-up visits, she noted a drastic improvement in dizziness, nausea, and left-sided sensitivity to sound and motion and complete resolution of eyebrow movement difficulties. Due to persistent right-sided pulsatile tinnitus and autophony, the patient underwent right SSCD repair approximately six weeks after left-sided repair. This time allowed for adequate recovery and healing. Right SSCD skull base, middle cranial fossa repair and secondary craniotomy were performed in a manner that mirrored the prior left repair. There were no complications. Post-operatively, the patient noted nausea, development of facial edema around cranial incision, and significant soreness of the right-side jaw. Although, jaw soreness was likely due to the surgical approach. During her six-week follow-up visit, the patient noted resolution of jaw pain. Additionally, she reported significant improvement in hyperacusis bilaterally, as well as improvement in her hearing and noise sensitivity on the right, which is now quiet.

## Discussion

Symptom overlap between comorbidities can make accurate diagnosis challenging. SSCD is characterized by fistula development between the superior semicircular canal and the middle cranial fossa [1-4]. EDS-HT is a genetic disorder resulting in collagen synthesis deficits [11,12]. In the former, patients experience vertigo, pulsatile tinnitus, conductive hearing loss, and headache [13]. In the latter, patients can also experience headache [14] and dizziness [15], but otological symptomology is rare [16]. The patient presented in this report, similar to our previously reported patients [10,11], exhibited symptoms characteristic of both SSCD and EDS-HT. Of note, although audiometry revealed an air-bone gap in the first case presented by our lab [11], this was not seen in the second case [10], nor the currently presented case. 

Ultimately, surgical repair in all patients resulted in symptomatic improvement on the ipsilateral side [10,11]. Although SSCD’s precise etiology remains unknown, a fragile temporal bone likely increases the risk of its development.

**Figure 3:**
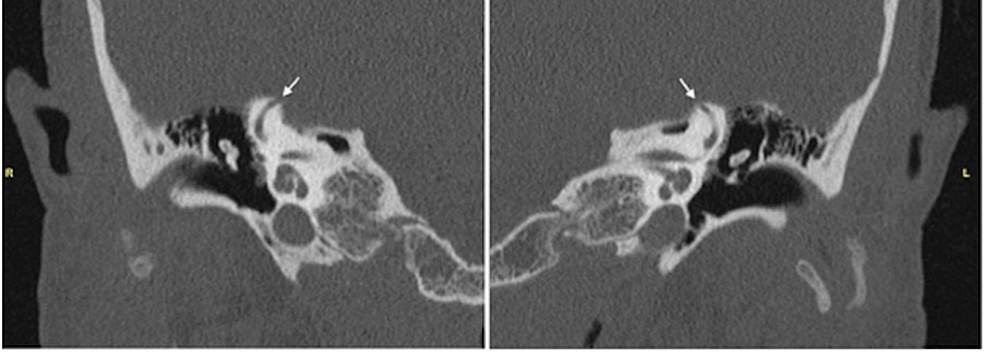
A 53-year-old woman presenting with migraines, vertigo, dizziness, and right-side greater than left-side tinnitus, hyperacusis, and chewing internal sound amplification High-resolution CT scans demonstrate area of 5 mm and 4 mm dehiscence of the (R) right and (L) left superior semicircular canal apices, respectively. Citation from Chung et al. [10] CT, computed tomography

**Figure 4:**
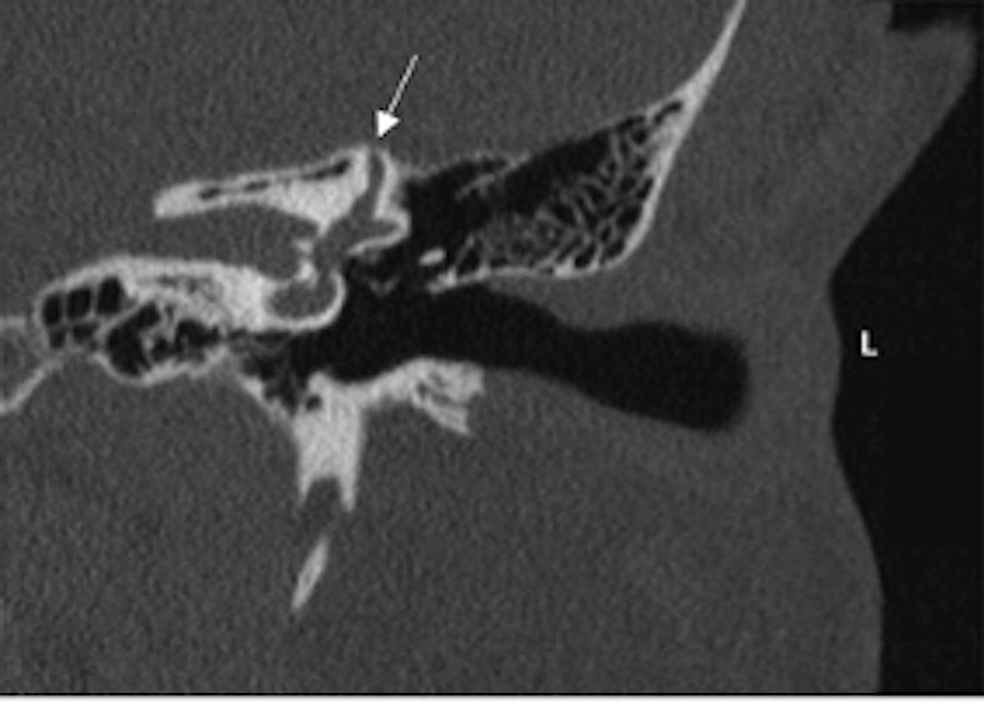
A 37-year-old woman presenting with hyperacusis, tinnitus, and vertigo. High-resolution CT scan of her left temporal bone shows her (L) left superior semicircular canal. The white arrow indicates thinning of arcuate eminence. Citation from Preet et al. [11] CT, computed tomography

Collagens are the most abundant proteins in mammals, composed of 28 members that contain at least one triple helical domain [17]. Type 1 collagen is formed by packing of polypeptide triple helices, providing load-bearing mechanical properties [18]. Since type 1 collagen is the main organic component of bone, patients with EDS-HT may develop a delicate temporal bone and have increased susceptibility to dehiscence via subsequent trauma, osteopenia, or elevated intracranial pressure [19]. As seen in this report, our patient began experiencing symptoms after a traumatic ice-skating fall. Thus, she presented an anecdotal case of SSCD symptoms occurring after trauma. Our prior reports [10,11], along with the patient in this study, presented with symptoms characteristic of both SSCD and EDS-HT and noted symptomatic improvement as well as MRI demonstrated structural resolution with surgical repair. Given this potential link, we postulate that EDS-HT may have predisposed our cohort to SSCD, as demonstrated by our previous work (Figures 3, 4) [10,11]. However, due to the rarity of both disorders, further research is needed to elucidate this potential connection. Interesting future investigations may further define the correlation between trauma, SSCD, and EDS-HT.

## Conclusions

In this case report, we presented the case of a female patient with EDS-HT who presented with bilateral SSCD. Due to its ability to confer bone fragility, we hypothesize that EDS-HT may augment the risk for SSCD development. Such findings call for further studies to evaluate the possible connection between these two pathologies.
